# Parenting in Moments: An Ecological Momentary Assessment of Parenting and Adolescent Substance Use and Externalizing Behaviors

**DOI:** 10.1111/sode.70095

**Published:** 2026-09-14

**Authors:** Tara M. Chaplin, Mary Ryan, Margaret F. Bedillion, Timothy W. Curby, Emily B. Ansell

**Affiliations:** 1 Department of Psychology, George Mason University, Fairfax, Virginia, USA; 2 Department of Psychiatry, Columbia University Irving Medical Center, New York, New York, USA; 3 Department of Biobehavioral Health, Penn State University, University Park, Pennsylvania, USA

**Keywords:** adolescence, ecological momentary assessment, emotion, externalizing, parenting, substance use

## Abstract

A large body of traditional survey and lab-based research has found that parenting behaviors are associated with risk for substance use (SU) and externalizing symptoms in adolescents. It is presumed that parenting effects on adolescent SU and symptoms unfold within families over moments in everyday life. However, this has rarely been tested. The present ecological momentary assessment (EMA) study tested within-person momentary associations among parenting, adolescent emotional and reward arousal, and SU and externalizing symptoms. Eighty-four 14–18 year olds completed five surveys per day for 28 days, reporting on parenting (negative parenting, parental warmth, and parental structure), negative emotional and reward arousal, and their SU and externalizing symptoms. Results of multi-level models found that momentary within-person increases in negative parenting and decreases in parental warmth were associated with greater within-person concurrent adolescent SU and next timepoint externalizing symptoms. Momentary within-person increases in negative parenting and decreases in warmth were also associated with within-person adolescent increased negative emotional arousal (particularly for girls) and blunted reward arousal. Momentary within-person increases in negative emotional arousal were associated with within-person increases in next timepoint externalizing symptoms, particularly for boys. Interestingly, findings for parental structure were different from other forms of parenting, with lower within-person structure associated with *lower* symptoms. Overall, this study extended prior between-person survey and laboratory research by identifying that parenting in moments in daily life may lead to arousal and momentary risk for adolescent substance use and acting out behaviors, supporting the development of momentary parenting interventions to prevent SU and symptoms.

## 1 | Introduction

Adolescence is a risk period for increases in substance use (SU) and externalizing symptoms (acting out behaviors, including talking back to adults and breaking rules) (Givens and Reid 2019; Miech et al. 2024). Adolescent SU and externalizing symptoms are correlated with one another, and both have significant consequences. They are associated with impaired driving, academic problems, and increased risk for psychological disorders into adulthood (e.g., Brook et al. 2002; Hinshaw 1992). Notably, the correlates of SU and externalizing symptoms may be different for girls and boys, but sex differences are understudied (Amaro et al. 2001).

Given this impact, it is important to identify risk factors that can be targeted in prevention programs. Prior traditional survey and laboratory studies have identified maladaptive parenting as a risk factor for SU and externalizing symptoms (e.g., Pinquart 2017). It is presumed that parenting effects on SU and symptoms occur within families over moments in real-world contexts. However, prior studies focus on between-person global parenting that is reported on over long time periods or observed in the lab environment. The present study adds to the field by using ecological momentary assessment (EMA) to examine if within-person momentary changes in parenting predict within-person adolescent SU and externalizing behaviors in daily life.

### 1.1 | Parenting and SU/Externalizing Symptoms

Theorists have proposed that parenting impacts child and adolescent social-emotional development and risk for SU and externalizing symptoms (Baumrind 1966; Morris et al. 2017; Pinquart 2017; Rusby et al. 2018). These theories suggest several unique forms of parenting behavior that each impact youth outcomes. Negative parenting (harsh, critical parenting) is theorized to act as a stressor for youth, leading to negative outcomes. Parental warmth (support, affection) is proposed to allow emotional security and adaptive regulation, reducing SU and externalizing symptom risk. Parental structure (setting rules, monitoring) is proposed to provide limits, protecting youth from interacting with risky peer contexts, decreasing SU and externalizing behavior risk. Consistent with theory, traditional survey and lab-based studies that test between-person associations have found that adolescents whose parents have higher mean levels of negative parenting, lower warmth, and lower structure have greater SU and externalizing symptoms (Pinquart 2017; Rusby et al. 2018).

### 1.2 | Emotion and Reward Arousal

One way that parenting may contribute to adolescent SU and externalizing symptoms is through impacts on adolescent arousal. Negative parenting behaviors and low warmth may lead to heightened adolescent negative emotional arousal (Morris et al. 2017). Youth may then seek out substances to down-regulate their negative arousal, consistent with self-medication theories (Koob and Le Moal 1997), or may express negative arousal as angry and defiant externalizing symptoms (Kim et al. 2007). Negative parenting, low warmth, and low structure can also strain (or fail to support) reward system development, leading to blunted reward arousal (Qu et al. 2015). Blunted reward arousal can lead adolescents to use substances or act out to upregulate reward arousal (Beauchaine 2012; Zuckerman 2007).

Consistent with theory, laboratory and functional magnetic resonance imaging (fMRI) studies that examined between-person associations have found that parents with higher mean negative parenting and lower warmth have adolescents with higher negative emotion-related arousal (Chaplin et al. 2012; Romund et al. 2016), arousal which is linked with greater adolescent SU and externalizing symptoms (e.g., Elsayed et al. 2018; Kim et al. 2007). Also, laboratory and fMRI studies have found that parents with higher mean negative parenting and lower warmth and structure have adolescents with altered (sometimes heightened, sometimes blunted) reward arousal (Holz et al. 2018; Kahn et al. 2015; Qu et al. 2015) and that altered reward arousal is associated with greater adolescent SU and externalizing symptoms (Bjork et al. 2010; Gatzke-Kopp et al. 2009; Gonçalves et al. 2022).

### 1.3 | EMA of Parenting, Arousal, and SU/Externalizing Symptoms

Here we propose, consistent with dynamic systems theories of development (e.g., Smith and Thelen 2003) that the parenting effects described above at a between-person level actually occur over multiple proximal interactions in daily life *within* families (Keijsers et al. 2022; Smyth and Heron 2014). Each small momentary parenting behavior (e.g., saying “good job”; yelling at an adolescent) may affect an adolescent’s arousal and choice to engage in SU and externalizing behaviors in the moment. This means that each momentary parenting choice may be an inflection point, with consequences for adolescent arousal and behaviors (Keijsers et al. 2022). Thus, even a parent who is globally low in warmth (at a between-person level) may in some moments (within person) offer warmth, which may proximally lead to lowered within-person adolescent negative emotional arousal and SU and externalizing behaviors. Examining parenting behavior only at a between-person level would mask this within-person impact.

EMA can test these within-person momentary associations (Russell and Gajos 2020; Smyth and Heron 2014). First, EMA can examine how parenting and arousal unfold in specific moments within families. It can test whether global aspects of parenting that matter for SU and symptoms at a between-person level are the same or different from proximal aspects of parenting that matter for outcomes within person (e.g., globally, high parental structure may matter for SU, but, proximally, parent yelling and criticism may be more important). For example, some theorists argue that the between-person association found between high structure/monitoring and low SU and symptoms reflects a generally close parent-youth relationship (and higher youth disclosure to parents), but in everyday moments *within* families, a parent setting rules on their adolescent may not lead their adolescent to decrease their acting out behaviors in the next moments or hours that day (Keijsers 2016). Consistent with this, one panel longitudinal study found that parental knowledge/monitoring was associated with SU at between-person, but not within-person, levels (Coley et al. 2008). Second, EMA tests how parenting, arousal, and SU/symptom associations unfold in real-life contexts, where parenting naturally occurs and closer in proximity to the experience. We assume parenting effects seen in the lab extend into real-life, but this is not known. Third, intensive assessment results in more accurate reporting of behaviors than global retrospective surveys, particularly for sensitive behaviors such as SU. Fourth, EMA can identify entry points for momentary interventions to change parenting (or adolescent arousal) in everyday moments to prevent SU and symptoms.

Despite this, no published EMA study has tested effects of parenting on SU or externalizing symptoms in adolescents. One EMA study found that momentary within-person parental heterosexism was associated with within-person increased substance craving in sexual minority adolescents (Parnes et al. 2023). One daily diary study showed that daily within-person increases in negative parenting (e.g., harsh punishment) and decreases in parental warmth (e.g., praising, spending positive time with child) were associated with greater within-person externalizing symptoms in 3–8-year-olds (Leijten et al. 2024), but there has not been work with adolescent externalizing symptoms.

EMA also can assess adolescent arousal in real life moments. A few initial EMA studies have examined parenting effects on adolescent arousal, finding that momentary within-person increases in negative parenting (e.g., conflict, criticism) and decreases in parental warmth (expressed warmth, connectedness, and cohesion) were associated with increased within-person negative emotional arousal (Bülow et al. 2022; Fosco and Lydon-Staley 2020; Janssen et al. 2024) and blunted positive emotional arousal (happiness, lively and engaged states—which may be associated with reward arousal) in adolescents (Bülow et al. 2022; Fosco et al. 2021; Fosco and Lydon-Staley 2020). And a few EMA studies found within-person associations between momentary increases in negative emotional arousal and drinking in adolescent heavy drinkers (Kauer et al. 2009; Padovano and Miranda 2021—but see Crooke et al. 2013 for null findings) and have found within-person associations between greater negative emotional lability and greater externalizing symptoms in children (Leaberry et al. 2017). A few EMA studies have found within-person associations between momentary increases in positive emotional arousal (including happiness, well-being, and also alertness, which could be related to reward arousal) and increases in adolescent SU and externalizing symptoms, with two studies finding blunted positive arousal associated with greater adolescent nicotine use and externalizing symptoms (Mereish et al. 2022; Scott et al. 2020) and two studies finding higher positive arousal associated with adolescent moderate drinking and cannabis use (Brick et al. 2023; Crooke et al. 2013). Overall, this initial EMA work supports that momentary within-person parenting may be associated with within-person adolescent arousal and outcomes in daily life; however, this literature is small, and more work is needed.

### 1.4 | Sex Differences

There may be sex differences in parenting associations with negative emotion and reward arousal. Theorists have proposed that adolescent girls may be more likely to respond to environmental stressors with high negative emotional arousal than boys, due to gender role socialization and biological factors, and that negative arousal is a stronger risk factor for SU and other outcomes for girls than boys (Chaplin and Aldao 2013; Hankin et al. 2007). Thus, the pattern noted above with parenting predicting negative emotional arousal and negative arousal predicting SU and externalizing symptoms may be more common for girls. Some fMRI research has found this at a between-person level (Chaplin et al. 2019). There are also sex differences in reward arousal, with boys showing higher reward arousal and sensation seeking than girls (Cross et al. 2013). Some theorists propose that reward arousal may be a stronger predictor of SU and externalizing symptoms for boys than girls (Beauchaine 2012; Chaplin et al. 2023). Thus, the pattern noted above with parenting predicting altered reward arousal and reward arousal predicting SU and symptoms may be more common for boys.

### 1.5 | The Present Study

The present study will extend the literature by using EMA to examine momentary associations in daily life among parenting, emotion and reward arousal, and SU and externalizing symptoms in adolescents (see Figure 1). First, we hypothesized that momentary within-person parenting behavior (higher negative parenting, lower parental warmth, and possibly parental structure) would be associated with within-person increases in adolescent SU and externalizing symptoms. Second, we hypothesized that momentary within-person parenting (higher negative parenting, lower warmth, and possibly structure) would be associated with within-person increases in negative emotional arousal (more so for girls) and alterations in reward arousal (more so for boys). Third, we hypothesized that higher momentary within-person negative emotional arousal (more so for girls) and altered within-person reward arousal (more so for boys) would be associated with momentary within-person increases in SU and externalizing symptoms.

## 2 | Method

### 2.1 | Participants

Participants were eighty-four 14–17-year-olds (with one 18-year-old), 37 boys and 47 girls (for additional demographic information, see Table 1). Adolescents were recruited from a larger study of parenting, adolescent fMRI responses, and SU to participate in an optional EMA component. For the larger study, adolescents and parent(s) were recruited from a suburban area in the mid-Atlantic United States. The larger study included adolescents who were 11–14 years old at baseline, safety-eligible for MRI, and English speaking, and excluded adolescents with intellectual disability, psychosis, or history of severe brain injury. The larger study and EMA component were approved by the University Institutional Review Board and informed parental consent and adolescent assent were obtained.

For the larger study, adolescents completed a lab and fMRI session at baseline and completed questionnaires at 1-, 2-, and 3-year follow-up sessions. Adolescents were invited to participate in the additional EMA once during the follow-up period. We prioritized recruiting adolescents to the EMA who had lifetime SU and/or high maladaptive parenting (based on reports in the larger study) to maximize the range of SU reporting during the 28-day EMA. One hundred and five adolescents agreed and began the EMA. Eighteen dropped out of the EMA after the first day and three had poor EMA compliance (<40%), resulting in a final analytic sample of 84 for the present paper. Of the 84 adolescents, 64 (76%) had lifetime SU and/or high maladaptive parenting (45 had lifetime SU, 33 had maladaptive parenting).

### 2.2 | EMA Procedure

Adolescents completed a 28-day EMA, with assessments five times per day. The first assessment was sent at 7 a.m. and completed upon wake up, the next three were sent at random times at least 1.5 h apart between 2:30 and 8:30 p.m. (after school), and the fifth was sent at 9:30 p.m. and completed before bedtime. Assessments were not sent during school hours because parenting and SU are not likely to occur during the school day. Adolescents were notified via mobile application alerts and text messages when an assessment was open. If not completed, reminders were sent after 5 and 15 min. Each assessment remained open for completion until the next assessment was sent. Similar assessment schedules are used in EMA studies with teens (Heron et al. 2017). The 28-day duration was chosen to balance sampling over a long enough period to capture SU with limiting burden on adolescents. Behavior that occurred after the bedtime assessment but before waking was reported in the waking survey.

Adolescents completed a 30-min training on the smart-phone EMA application (Metric Wire). Study staff monitored adolescents’ EMA compliance daily. If there was low compliance or data irregularities, staff contacted participants to provide support. Adolescents were compensated $1 for each assessment completed and a $25 bonus for each week EMA compliance was ≥85%.

### 2.3 | EMA Measures

#### 2.3.1 | Parenting

Adolescents reported the level of three parenting behaviors they experienced (from any parent) since the last assessment on a 0–100 visual analogue scale (VAS). Negative parenting was measured with five items (my parent… criticized me; yelled or spoke harshly to me; punished me; got mad or annoyed at me; was annoying). Parental warmth was measured with five items (my parent… showed me love; got along well with me; listened to me; my parent and I had fun; I felt close to my parent). Parental structure was measured with two items (my parent… stated or explained a rule; knew where I was and/or who I was with). Items were based on prior EMA studies examining parenting (Bülow et al. 2022) and/or a well-established parenting questionnaire (Dadds et al. 2003). Items were averaged to create parenting variables.

#### 2.3.2 | Emotional Arousal

Adolescents reported their level of negative emotional arousal currently (“right now”) on a 0–100 VAS using five items (sad; mad or annoyed; nervous; upset; stressed). Items were adapted from the Positive and Negative Affect Schedule—Child Form (Laurent et al. 1999) and have been used in EMAs with adolescents (e.g., Bülow et al. 2022). Items were averaged to create a negative emotion score.

#### 2.3.3 | Reward Arousal

Adolescents reported their level of reward arousal currently (“right now”) on a 0–100 VAS with four items (I crave excitement and new sensations; I want to try something new; I want to try something fun; I feel excited). Items were adapted from the Behavioral Activation Scale (Carver and White 1994), a widely used measure of reward system sensitivity. Items were averaged to create a negative emotion score.

#### 2.3.4 | Substance Use

Adolescents reported whether they used alcohol, nicotine, cannabis, and/or any other drug since the last assessment (yes/no). Adolescents could endorse more than one substance.

#### 2.3.5 | Externalizing Symptoms

Adolescents reported on their externalizing symptoms since the last assessment on a 0–100 VAS with four items (do something your parents don’t like; put off something your parent asked you to do; talk back, roll your eyes, or disagree with an adult; do something behind your parent’s back). Items were adapted from an EMA study of adolescents (Li and Lansford 2018) and a widely-used survey (Gadow et al. 2002). Items were averaged to create an externalizing symptom variable.

### 2.4 | Analysis Plan

To test hypotheses, we estimated a series of two-level multilevel models (MLMs) (with repeated timepoints nested within participants) using R software, with the lme function for continuous outcomes and glmer function for the categorical SU outcome (analysis code available at [masked for review]). An autoregressive covariance structure was used to account for potential autocorrelation in the repeated measures. Models partitioned the variance of the predictor variable into within- and between-level components. Predictor variables were centered.

Models controlled for study day (to control for passage of time) and weekend status at level 1. We considered adolescent sex and age as additional covariates. Adolescent sex was conceptually viewed as a moderator of effects of parenting on arousal and effects of arousal on SU/externalizing symptoms, and thus sex is included in those models. Age was tested as a predictor of the outcome variables (SU, externalizing symptoms, arousal) in MLMs and did not predict these. Thus, age was not included as a covariate in final models for parsimony.

To test Hypothesis 1 (momentary within-person parenting associations with within-person SU and externalizing symptoms), we conducted MLMs. Each MLM examined within-person parenting at the same timepoint (concurrent) and within-person parenting behaviors at the prior timepoint (lagged) predicting within-person SU or externalizing symptoms. The concurrent terms test associations at the same timepoint. The lagged terms test if parenting *precedes* SU and externalizing symptoms, which can show directionality. Each of the three parenting variables (negative parenting, warmth, structure) and the two outcome variables (SU, externalizing symptoms) were examined in separate MLMs (six total models).

To test Hypothesis 2 (momentary within-person parenting would be associated with within-person higher negative emotional arousal [more so for girls] and altered reward arousal [more so for boys]), MLMs were conducted with within-person parenting at the same timepoint, sex, and a within-person parenting by sex cross-level interaction predicting within-person emotion/reward arousal. Each of the three parenting variables (negative parenting, warmth, structure) and the two outcomes (negative emotion, reward) were examined in separate MLMs (six models). These models tested same-timepoint associations between parenting and emotion/reward arousal only and not lagged because the wording for parenting was “since last survey” and the wording for arousal was “right now.” Because of this language, responses at the same timepoint reflect associations between recent parenting and current emotion/reward arousal.

To test Hypothesis 3 (momentary within-person higher negative emotional arousal [more so for girls] and altered reward arousal [more so for boys] will be associated with within-person increases in SU and externalizing symptoms), MLMs were conducted with within-person arousal at the same timepoint (concurrent), within-person arousal at the prior timepoint (lagged), sex at Level 2, and within-person arousal concurrent by sex and within-person arousal lagged by sex cross-level interactions predicting within-person SU/externalizing symptoms. Each of the two arousal variables (negative emotion, reward) and the two outcomes (SU, externalizing symptoms) were examined in separate MLMS (four models). For significant interactions, simple slopes analyses were conducted with R software.

For those situations where a parenting variable had a significant effect on arousal and arousal had a significant lagged effect on SU or externalizing symptoms, in the same direction, we conducted exploratory mediation analyses (reported in the Supporting Information file, see Supplementary Tables 1 and 2).

## 3 | Results

### 3.1 | Preliminary Analyses

Measure reliabilities are in the Supporting Information file. Participants’ mean compliance in completing EMA assessments across the 28-day EMA was 75%, similar to other adolescent EMA studies (Wen et al. 2017). Continuous variables were examined for outliers. Negative parenting and externalizing symptoms had outlier cases, and those were winsorized (set to equal two *SD*s above the mean). Following winsorization, all variables included in analyses had acceptable skewness and kurtosis (values < 2).

Descriptive information on the variables is reported in Table 1. For SU, there were 177 SU reports, with 113 cannabis, 58 nicotine, and 18 alcohol (adolescents could report more than one substance per SU episode). Intraclass correlation coefficients (ICCs) were 0.57 for externalizing symptoms, 0.52 for negative emotional arousal, 0.53 for reward arousal, 0.51 for negative parenting, 0.58 for warmth, and 0.53 for structure, indicating that a substantial proportion of the variance (42%–49%) was due to moment-to-moment changes within person. For SU, since the MLM was logistic, information was not available to calculate an ICC.

### 3.2 | Parenting Associations With SU and Externalizing Symptoms

Findings are presented in Table 2.

#### 3.2.1 | SU

Within-person negative parenting was associated with concurrent, but not lagged, within-person SU. In moments when reported negative parenting was higher than an adolescent’s average level, adolescents were more likely to report using substances in the same interval. Within-person parental warmth and structure were not associated with SU. Between-person effects of parenting and effects of weekend status and study day were not significant.

#### 3.2.2 | Externalizing

Within-person negative parenting was associated with concurrent and lagged within-person externalizing symptoms. In moments when reported negative parenting was higher than an adolescent’s average level, adolescents reported increased externalizing symptoms in the same and in subsequent intervals. The between-person effect of negative parenting was also significant.

Within-person parental warmth was associated with lagged, but not concurrent, within-person externalizing symptoms. In moments when reported parental warmth was lower than an adolescent’s average level, adolescents reported increased externalizing symptoms in subsequent intervals. The between-person effect of warmth was not significant.

Within-person parental structure was associated with concurrent, but not lagged, within-person externalizing symptoms. Unexpectedly, in moments when reported parental structure was lower than an adolescents’ average level, adolescents reported *decreased* externalizing symptoms in the same interval. The between-person effect of structure was not significant.

In the above models, effects of weekend status and study day were not significant.

### 3.3 | Parenting Associations With Arousal (by Sex)

Findings are presented in Table 3.

#### 3.3.1 | Negative Arousal

There was a significant cross-level within-person negative parenting by sex interaction effect on negative emotional arousal. Higher within-person negative parenting predicted higher within-person negative emotional arousal for girls (*b* = 0.34, *SE* = 0.02, *p* < 0.001) and boys (*b* = 0.26, *SE* = 0.02, *p* < 0.001), with a larger coefficient for girls (see Figure 2a). At times when adolescents recently experienced higher than average negative parenting, adolescents—especially girls—reported increased negative emotional arousal. There were also within-person main effects of higher negative parenting on higher negative emotional arousal. The between-person effect of negative parenting was significant.

There was a significant cross-level within-person parental warmth by sex interaction effect on negative emotional arousal. Lower within-person warmth predicted higher within-person negative emotional arousal for girls (*b* = −0.08, *SE* = 0.01, *p* < 0.001) but not boys (*b* = 0.00, *SE* = 0.01, *p* = 0.72) (Figure 2b). At times when adolescents recently experienced lower than average parental warmth, girls, but not boys, reported increased negative emotional arousal. There was also a within-person main effect of lower parental warmth on higher negative emotional arousal. The between-person effect of warmth was not significant.

The cross-level parental structure by sex interaction effect on negative emotion was not significant. There was a within-person main effect of structure. Unexpectedly, in moments when reported parental structure was lower than an adolescent’s average level, adolescents reported *decreased* negative emotional arousal. The between-person effect of structure was not significant.

In the above models, there were significant effects of sex, with girls reporting higher negative arousal than boys and weekend, with adolescents reporting lower negative arousal on weekends. The effect of study day was not significant.

#### 3.3.2 | Reward Arousal

There was not a significant cross-level within-person negative parenting by sex interaction predicting reward arousal. There was a within-person main effect of negative parenting. In moments when an adolescent recently experienced higher than average negative parenting, they reported blunted reward arousal. The between-person negative parenting effect was not significant.

There was a significant cross-level within-person parental warmth by sex interaction predicting reward arousal. Lower within-person parental warmth predicted blunted within-person reward arousal for girls (*b* = 0.24, *SE* = 0.01, *p* < 0.001) and for boys (*b* = 0.18, *SE* = 0.02, *p* < 0.001), with a larger coefficient for girls. At times when adolescents recently experienced lower than average parental warmth, adolescents—especially girls—reported blunted reward arousal. There was also a within-person main effect of lower warmth on blunted reward arousal. The between-person effect of warmth was significant.

There was not a significant cross-level parental structure by sex interaction predicting reward arousal. There was a within-person main effect of structure. In moments when an adolescent recently experienced lower than average parental structure, they reported blunted reward arousal. The between-person effect of structure was significant.

In the above models, there were significant effects of sex, with boys reporting higher reward arousal than girls, of weekend, with adolescents reporting higher reward arousal on weekends, and of study day, with higher reward arousal in later days.

### 3.4 | Arousal Associations With SU/Externalizing Symptoms (by Sex)

Findings are presented in Table 4.

#### 3.4.1 | SU

For negative emotional arousal associations with SU, the MLM that included the lagged terms failed to converge. We removed the lagged terms and re-ran the model to examine concurrent associations. There was a significant concurrent cross-level within-person negative emotional arousal by sex interaction effect on SU. *Lower* within-person negative emotional arousal was associated with greater within-person concurrent SU for boys (*b* = −0.06, *SE* = 0.02, *p* = 0.01), but not for girls (*b* = −0.01, *SE* = 0.01, *p* = 0.48). Because of the wording of SU as “since last survey” and emotional arousal as “right now,” these concurrent findings indicate that, at times when boys recently used substances, they reported decreased negative emotional arousal.

For reward arousal to SU, the MLM that included the lagged terms failed to converge. We removed the lagged terms and examined concurrent associations. There was a significant concurrent cross-level within-person reward arousal by sex interaction effect on SU. Higher within-person reward arousal was associated with greater within-person concurrent SU for girls (*b* = 0.03, *SE* = 0.01, *p* < 0.001) and lower within-person reward arousal was associated with greater within-person concurrent SU for boys (*b* = −0.03, *SE* = 0.02, *p* = 0.03). At times when they recently used substances, girls reported increased and boys reported blunted reward arousal. There was also a main effect of higher within-person reward arousal on greater within-person concurrent SU.

In the above models, the between-person effects of arousal and effects of sex, weekend status and study day were not significant.

#### 3.4.2 | Externalizing Symptoms

For negative emotional arousal, there was a significant cross-level within-person arousal by sex interaction on lagged externalizing symptoms. Higher within-person negative emotional arousal predicted higher within-person lagged externalizing symptoms for boys (*b* = 0.13, *SE* = 0.01, *p* < 0.001) and for girls (*b* = 0.06, *SE* = 0.01, *p* < 0.001), with a larger coefficient for boys (Figure 3). In moments when reported negative emotional arousal was higher than an adolescent’s average level, adolescents—particularly boys—reported increased externalizing symptoms in the subsequent interval. The main effect of higher within-person negative emotional arousal on higher within-person symptoms was significant for concurrent symptoms and a trend for lagged symptoms. The between-person effect of negative arousal was significant.

For reward arousal, there was not a significant cross-level within-person reward by sex interaction effect on externalizing symptoms. There was a main effect of higher within-person reward arousal on higher within-person concurrent (but not lagged) symptoms, such that, when an adolescent recently engaged in higher than average externalizing behaviors, they reported higher reward arousal. The between-person effect of reward arousal was not significant.

In the above models, effects of weekend status and study day were not significant. The main effect of sex was significant in one model, with boys reporting greater symptoms than girls.

## 4 | Discussion

The present study was one of the first to examine momentary within-person associations among parenting, arousal, and adolescent SU and externalizing symptoms in everyday life. Findings suggest that effects of negative parenting and low parental warmth on SU and externalizing risk found in prior traditional survey and laboratory studies at the between-person level may also be found at the within-person momentary level (and that effects of parental structure may operate differently at the momentary level). The study also examined adolescent arousal, finding sex-differentiated momentary associations with parenting and externalizing symptoms. Overall, findings support that parenting in everyday moments and associated adolescent arousal have important immediate consequences for behavior and may be viable targets for mobile health just-in-time interventions.

### 4.1 | Parenting and SU/Externalizing Symptoms

The present study found evidence that within-person momentary increases in negative parenting and decreases in warmth were associated with within-person momentary decisions to engage in substance use (in the same moments) and in externalizing behaviors (in the next moments). This is consistent with prior between-person survey and lab/fMRI study findings that parents with higher mean levels of harsh/negative parenting and lower warmth have adolescents with higher externalizing symptoms and greater SU (Pinquart 2017; Rusby et al. 2018) and extends these to within-person associations in real-world moments. These findings suggest that, if a parent increases their negative parenting or decreases their warmth in the moment, their adolescent immediately is sensitive to this and reacts by acting out (through SU or externalizing behaviors).

These within-person momentary findings (particularly the lagged effects) provide some evidence for a proximal causal role for parenting leading to adolescent behavior, given that within-person data can more precisely test causal processes (Keijsers 2016). In addition, these results support, for the first time, that parenting effects exist not only in the lab and in retrospective surveys over long periods, but also in real-life moments, where parenting naturally occurs. These findings suggest that just-in-time interventions can prompt parents in the moment to change their negative parenting or warmth to proximally reduce externalizing symptoms and SU in real-world contexts. Such momentary interventions could be added to traditional parenting interventions, which are delivered one time outside of real-world settings, boosting their effects (Smyth and Heron 2014).

Interestingly, the present study’s findings for within-person parental structure were different from between-person studies, highlighting the importance of conducting within-person parenting research. Prior between-person studies have found that parents with higher mean structuring (monitoring, rule-setting) have adolescents with lower mean SU and externalizing symptoms (e.g., Pinquart 2017). The present study, however, found that higher momentary within-person structuring was associated with *higher* within-person SU and externalizing behaviors (concurrently). This suggests that, in moments in daily life, increasing rule setting and monitoring by parents is associated with adolescents acting out *more*. While having clear rules and monitoring processes overall at a baseline level may be helpful, *in the moment*, it may be more important for parents to pause on rule setting and instead to show high warmth and low negative parenting, which may increase momentary closeness, decrease stress, and prevent acting out.

### 4.2 | Parenting, Negative Arousal, and SU/Externalizing Symptoms

The present study also sought to examine momentary adolescent arousal, processes that may be associated with parenting and SU and externalizing symptoms. The study found that recent higher negative parenting and lower parental warmth predicted momentary increases in negative emotional arousal (with a stronger prediction for girls) and that higher momentary negative emotional arousal predicted higher next timepoint externalizing symptoms (with a stronger prediction for boys). This suggests that momentary increases in negative parenting and decreases in warmth may trigger negative emotional arousal in the moment, and that negative arousal may lead adolescents to act out, possibly as a way to feel better (by engaging in exciting rule breaking behaviors) or as a way to express their negative emotions (by engaging in angry or defiant behaviors). Given this finding, momentary interventions can reduce negative parenting and warmth in the moment and/or can help adolescents regulate negative arousal during challenging parenting moments to decrease their acting out behaviors.

Notably, momentary increases in negative parenting predicted within-person increases in negative emotional arousal more strongly for girls than boys (consistent with prior between-person research—e.g., Chaplin et al. 2023). This suggests that girls are more likely to react to parenting in the moment by experiencing and focusing on their negative emotional arousal. It is interesting that parenting predicted negative emotional arousal more strongly for girls, but that this arousal predicted externalizing symptoms more strongly for boys. This may be due to a greater tendency to engage in acting out behaviors in boys. Girls’ momentary negative emotional arousal responses to parenting may lead to other outcomes. For example, theorists have proposed that girls’ pattern of high negative emotional arousal responses to stress may help explain girls’ greater internalizing symptoms than boys (Hankin et al. 2007). Thus, momentary effects of parenting in increasing girls’ negative arousal may lead to momentary feelings of depression or anxiety, and future EMA studies can examine this. Momentary negative arousal might also predict SU more strongly for girls than boys (Chaplin et al. 2019). Unfortunately, due to low occurrences of SU, there were convergence issues in the lagged models, and so we were unable to test if negative arousal predicted next-timepoint SU more so for girls.

### 4.3 | Parenting, Reward Arousal, and SU/Externalizing Symptoms

In the present study, higher momentary within-person negative parenting, lower parental warmth, and lower parental structure were associated with within-person blunted adolescent reward arousal. This is consistent with recent EMA studies showing that higher momentary maladaptive parenting is associated with lower momentary positive emotions (e.g., Bülow et al. 2022) and extends this specifically to reward arousal. Experiencing repeated moments of maladaptive parenting and subsequent decreases in reward arousal in daily life may be problematic, leading youth to disengage from the family and positive activities. Though we proposed that this would lead to externalizing symptoms, we did not find this association. Instead, blunted momentary reward arousal could lead to other outcomes, such as depressive symptoms. Blunted momentary reward arousal could also lead to using substances to up-regulate reward systems, as we proposed. The present study’s lagged models predicting SU did not converge, but there was a finding that blunted reward arousal was associated with concurrent SU for boys. Future EMA studies with greater ranges of SU could further examine this association.

### 4.4 | Summary

This study innovatively examined momentary within-person real-world associations among parenting, arousal, and SU and externalizing symptoms. It had several strengths, including intensive repeated assessments in real-world contexts and examination of momentary arousal and sex differences. However, there were some limitations. Adolescents were selected as higher risk for SU (due to lifetime SU or maladaptive parenting), and thus findings may not generalize to a low-risk community sample. Also, despite being recruited for elevated SU risk, there were a limited number of SU reports. This restricted power and may have led to convergence issues in the lagged SU models. In addition, the study was limited to adolescent reports, and future EMA research could add parent reports or passive sensing. Also, future EMA research can examine transactional associations between parenting and behavior.

Overall, the present study expanded understanding of parenting effects by establishing that negative parenting and low warmth may be associated with SU and externalizing symptoms within families in everyday moments in real-world contexts. The present study also suggests a potential role for negative emotional and reward arousal, which can be tested as potential mediators in future studies with larger samples. Findings suggest that parenting behavior and youth arousal may be viable targets for momentary interventions to reduce adolescent SU and externalizing symptoms.

## Supplementary Material

supplementary material **Supporting Table S1**: Parenting Associations with Adolescent Arousal (Mediation A Path) **Supporting Table S2**: *Negative Arousal Predicting Externalizing Symptoms, Controlling for Parenting (Mediation B Path)*

Additional supporting information can be found online in the Supporting Information section.

## Figures and Tables

**Figure 1:**
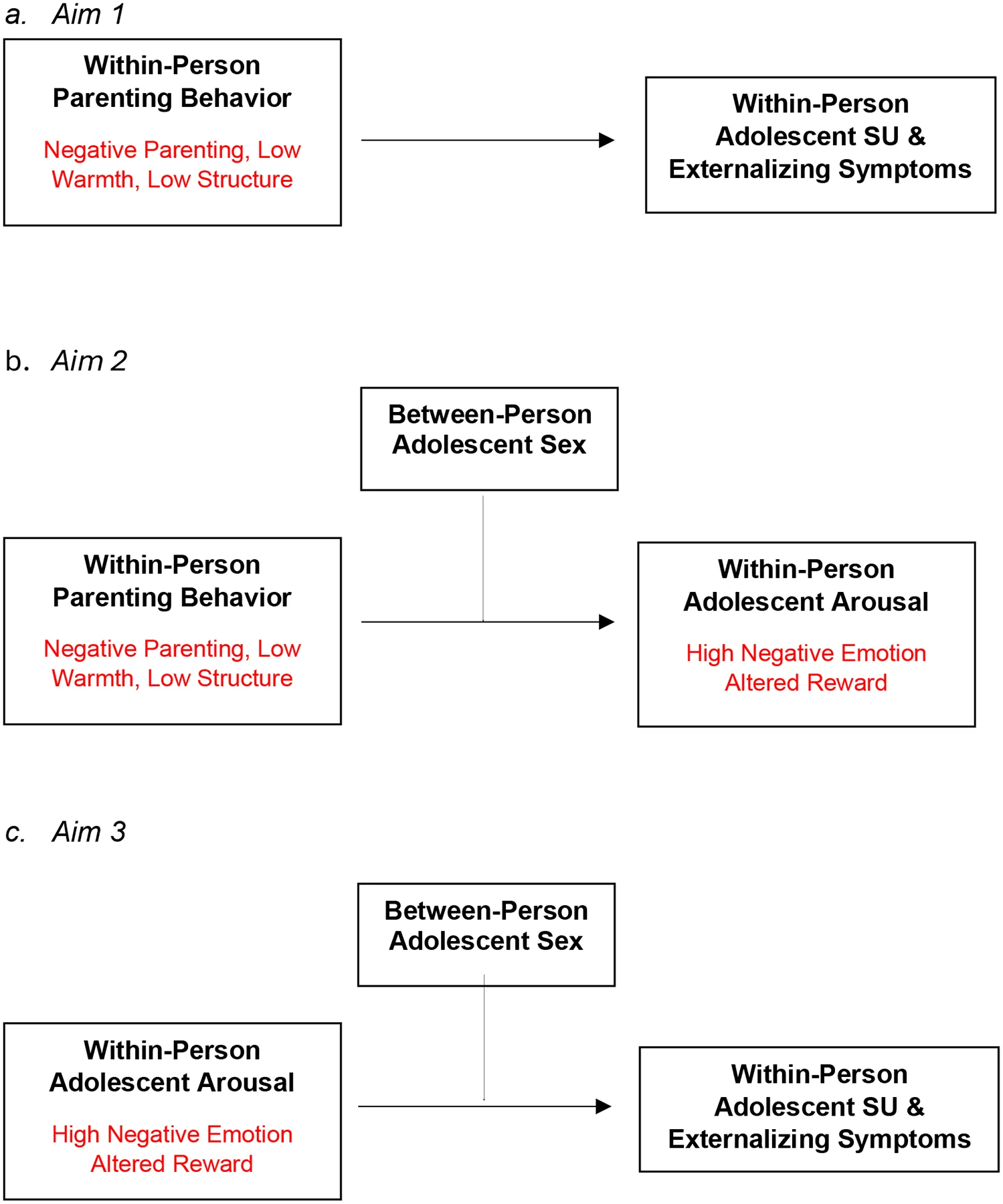
Conceptual Model Note. SU = substance use

**Figure 2:**
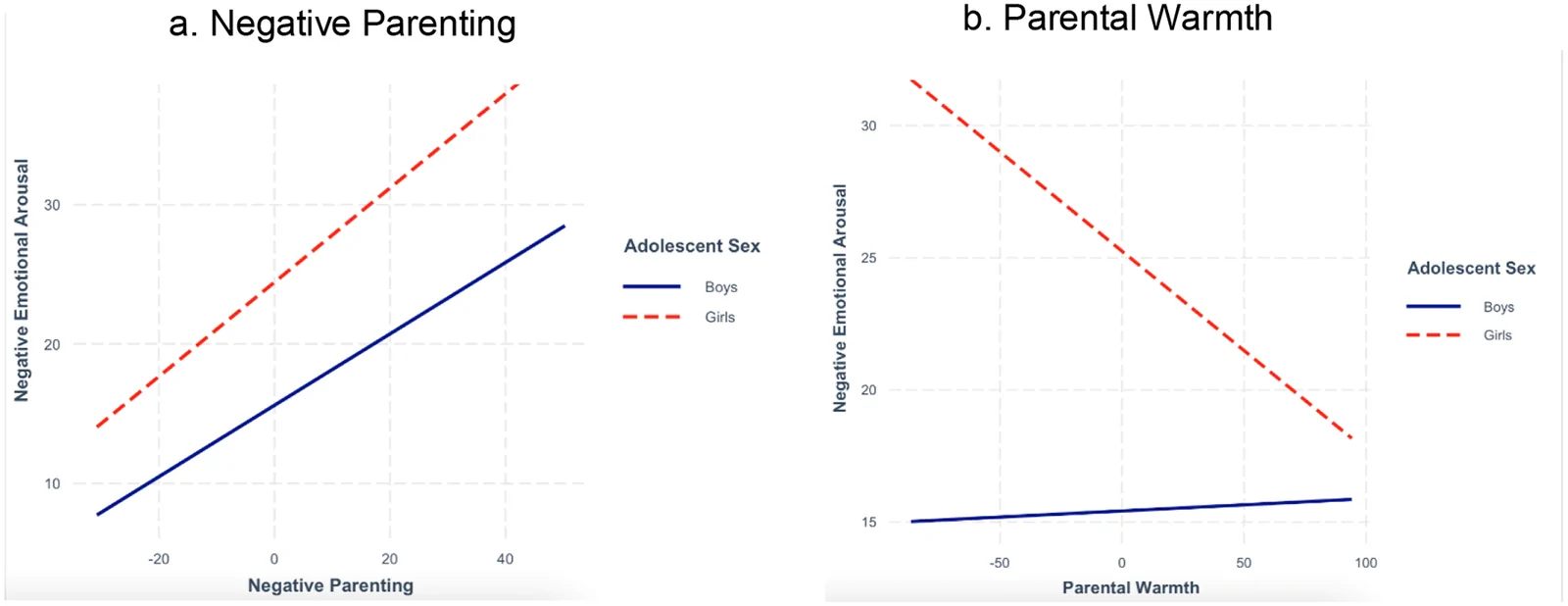
Interaction Simple Slope Plots for Within-Person Parenting to Negative Emotional Arousal by Sex

**Figure 3:**
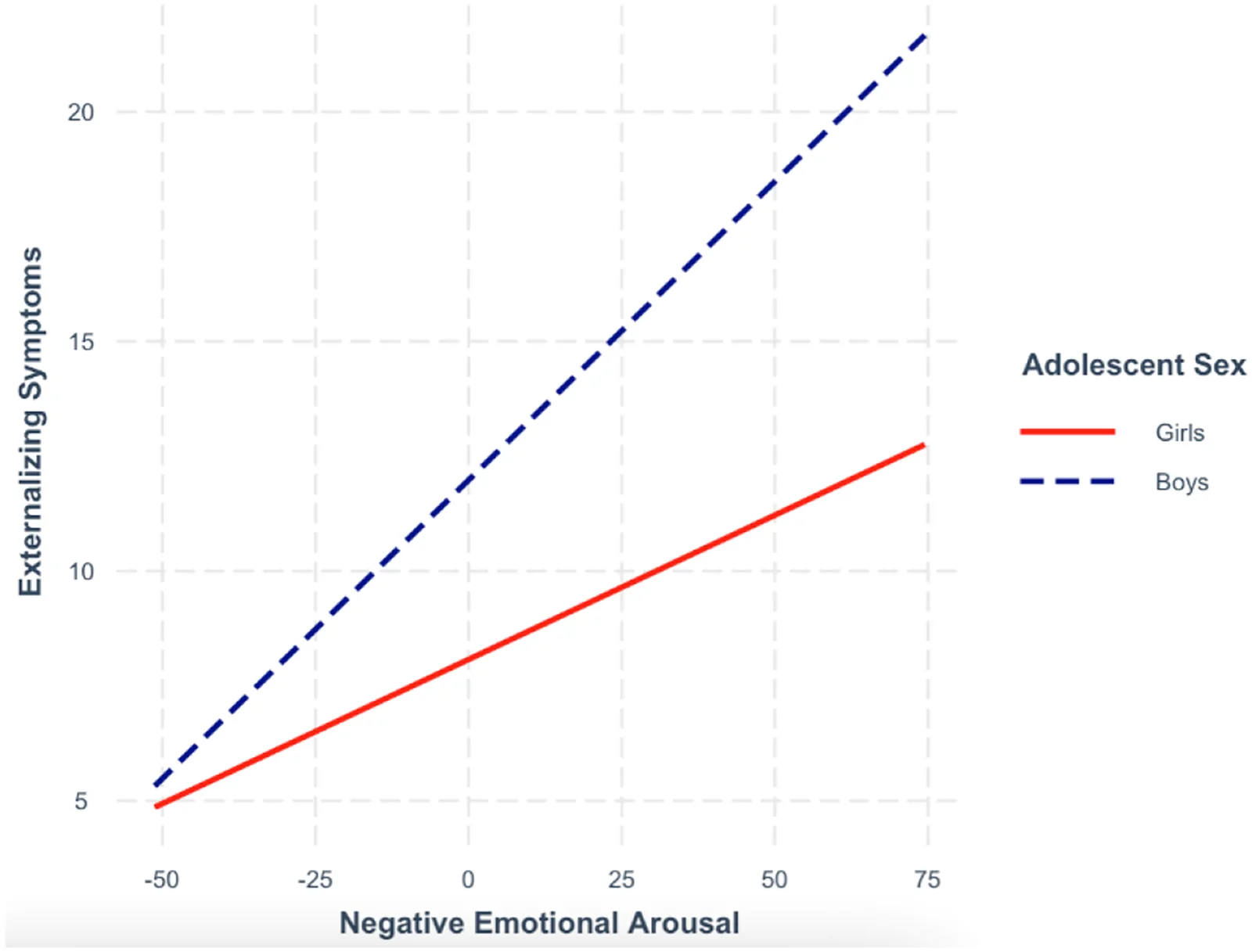
Interaction Simple Slope Plot for Within-Person Negative Emotional Arousal to Lagged Externalizing Symptoms by Sex

**TABLE 1 |:** Demographic information and descriptives.

	Mean (or %)	*SD*
Demographic variables:		
Adolescent age (years)	15.18	0.96
Adolescent race		
White	66.6%	
More than 1 race	19.0%	
Black	6.0%	
Asian	6.0%	
American Indian/Alaska Native	1.2%	
Other race	1.2%	
Adolescent ethnicity		
Hispanic	20.2%	
Non-Hispanic White	56.0%	
Yearly household income		
<$25,000	1.2%	
$25,00–$59,999	3.7%	
$60,000–$74,999	6.1%	
$75,000–$99,999	15.9%	
$100,000–$124,999	15.9%	
$125,000–$149,999	11.0%	
$150,000–$175,000	13.4%	
>$175,000	32.9%	
EMA variables:		
Negative parenting	10.93	12.12
Parental warmth	54.77	26.27
Parental structure	47.20	21.18
Negative emotional arousal	20.54	18.19
Externalizing symptoms	9.81	10.91
Substance use occasions	177	

*Note*:Race/ethnicity and income are similar to the local community, as planned.

**TABLE 2 |:** Parenting associations with adolescent SU and externalizing symptoms.

	Substance use			Externalizing symptoms		
	B	*SE*	*p*	b	*SE*	*p*
Negative parenting models						
Intercept	−12.01	1.53	<0.001	10.13	0.55	<0.001
Weekend status	0.56	0.30	0.06	−0.07	0.21	0.73
Study day	−0.004	0.02	0.84	−0.01	0.01	0.58
Neg parenting (between-person)	0.13	0.10	0.20	**0.89**	**0.07**	**<0.001**
Neg parenting (within-person, concurrent)	**0.03**	**0.01**	**0.01**	**0.29**	**0.01**	**<0.001**
Neg parenting (within-person, lagged)	0.01	0.01	0.47	**0.04**	**0.01**	**<0.001**
Parental warmth models						
Intercept	−12.15	1.41	<0.001	10.12	1.07	<0.001
Weekend	0.54	0.30	0.07	0.05	0.24	0.84
Study day	−0.01	0.02	0.71	−0.02	0.01	0.13
Warmth (between-person)	−0.03	0.04	0.50	−0.05	0.05	0.31
Warmth (within-person, concurrent)	0.01	0.01	0.25	0.001	0.01	0.81
Warmth (within-person, lagged)	0.005	0.01	0.57	−**0.02**	**0.01**	**0.01**
Parental structure models						
Intercept	−12.14	1.37	<0.001	10.17	1.09	<0.001
Weekend status	0.51	0.29	0.08	−0.03	0.23	0.89
Study day	−0.004	0.02	0.81	−0.02	0.01	0.22
Structure (between-person)	0.001	0.05	0.99	0.03	0.07	0.68
Structure (within-person, concurrent)	−0.002	0.01	0.80	**0.07**	**0.01**	**<0.001**
Structure (within-person, lagged)	−0.003	0.01	0.72	0.001	0.01	0.89

*Note*:Significant effects of interest are bolded. Abbreviation: Neg, negative.

**TABLE 3 |:** Parenting associations with adolescent arousal, by sex.

	Negative emotion arousal			Reward arousal		
	b	*SE*	*p*	b	*SE*	*p*
Negative parenting models						
Intercept	25.30	1.33	<0.001	33.79	2.62	<0.001
Weekend status	−0.93	0.36	0.01	1.69	0.49	0.001
Study day	0.03	0.02	0.25	0.11	0.03	0.001
Adolescent sex	−8.85	2.02	<0.001	8.18	3.98	0.04
Neg parenting (between-person)	**1.00**	**0.12**	**<0.001**	0.25	0.23	0.29
Neg parenting (within-person)	**0.34**	**0.02**	**<0.001**	−**0.05**	**0.02**	**0.04**
Neg parenting (w/in) × Sex	−**0.12**	**0.03**	**<0.001**	−0.02	0.04	0.69
Parental warmth models						
Intercept	25.50	1.82	<0.001	33.65	2.49	<0.001
Weekend status	−0.77	0.37	0.04	1.54	0.48	0.001
Study day	0.02	0.02	0.44	0.13	0.03	<0.001
Adolescent sex	−9.89	2.74	0.001	8.07	3.76	0.03
Warmth (between-person)	−0.04	0.06	0.49	**0.27**	**0.09**	**0.002**
Warmth (within-person)	−**0.10**	**0.01**	**<0.001**	**0.21**	**0.01**	**<0.001**
Warmth (w/in) × Sex	**0.08**	**0.02**	**<0.001**	−**0.07**	**0.02**	**<0.001**
Parental structure models						
Intercept	25.57	1.82	<0.001	34.16	2.54	<0.001
Weekend status	−0.94	0.37	0.01	1.62	0.49	0.001
Study day	0.02	0.02	0.33	0.12	0.03	<0.001
Adolescent sex	−9.89	2.74	0.001	6.75	3.83	0.08
Structure (between-person)	0.06	0.09	0.48	**0.32**	**0.12**	**0.01**
Structure (within-person)	**0.05**	**0.01**	**<0.001**	**0.06**	**0.01**	**<0.001**
Structure (w/in) × Sex	0.005	0.02	0.80	−0.02	0.02	0.46

*Note*:Significant effects of interest are bolded. Abbreviations: Neg, negative; W/in, within-person.

**TABLE 4 |:** Arousal predicting SU and externalizing symptoms, by sex.

	Substance use			Externalizing symptoms		
	b	*SE*	*p*	b	*SE*	*p*
Negative emotion models						
Intercept	−10.21	1.67	<0.001	8.07	1.16	<0.001
Weekend status	0.31	0.23	0.17	0.10	0.23	0.66
Study day	0.0002	0.01	0.99	−0.02	0.01	0.13
Sex	0.84	1.83	0.65	3.95	1.75	0.03
Negative emotion (between-person)	0.11	0.08	0.16	**0.44**	**0.07**	**<0.001**
Negative emotion (within-person, concurrent)	−0.01	0.01	0.48	**0.06**	**0.01**	**<0.001**
Negative emotion (within-person, lagged)	NC	NC	NC	0.02	0.01	0.08
Negative emotion (w/in, concurrent) × Sex	−**0.05**	**0.02**	**0.03**	**0.05**	**0.02**	**0.001**
Negative emotion (w/in, lagged) × Sex	NC	NC	NC	**0.03**	**0.02**	**0.05**
Reward models						
Intercept	−11.56	2.52	<0.001	10.01	1.48	<0.001
Weekend status	0.23	0.23	0.31	−0.03	0.24	0.90
Study day	−0.01	0.01	0.51	−0.02	0.01	0.16
Sex	−1.23	4.66	0.79	0.09	2.25	0.97
Reward (between-person)	0.02	0.15	0.89	0.06	0.06	0.32
Reward (within-person, concurrent)	**0.03**	**0.01**	**<0.001**	**0.02**	**0.01**	**0.03**
Reward (within-person, lagged)	NC	NC	NC	−0.001	0.01	0.95
Reward (w/in, concurrent) × Sex	−**0.06**	**0.02**	**<0.001**	−0.004	0.01	0.76
Reward (w/in, lagged) × Sex	NC	NC	NC	−0.01	0.01	0.55

*Note*:Significant effects of interest are bolded. Abbreviations: NC, did not converge; W/in, within-person.

## Data Availability

De-identified raw data from this study are available on request from the corresponding author under a data sharing agreement. The data are not publicly available due to privacy and sensitive nature of the data (substance use in adolescents).
