# Supplementary material for: Parenting in Moments: An Ecological Momentary Assessment of Parenting and Adolescent Substance Use and Externalizing Behaviors
Source: Soc Dev. Author manuscript; Available in PMC 2026 Oct 2. (PMC13626704; doi:10.1111/sode.70095)
Supplement: Supplementary file 1 [file NIHMS2210786-supplement-supplementary_material.docx]

**Supporting Information**

**Reliability**

We calculated multilevel reliability of the composite EMA variables using R software with the mlr function (following Tomko et al. 2014). Between-person reliability (generalizability of between person differences in the scale averaged across time, with time nested within people) was excellent (RkRn’s = 0.99 to 1.00). Within-person reliability (generalizability of within person variations in the scales averaged over items, with time nested within people), was acceptable (Rcn’s = 0.79 for negative parenting, 0.83 for warmth, 0.63 for negative arousal, 0.71 for reward arousal, and 0.63 for externalizing symptoms). Of note, lower within-person than between-person reliability is expected given that mood/behavior changes from moment to moment in intensive longitudinal studies (Tomko et al. 2014). Reliability for structure was low. Given this, analyses were conducted with the two structure items separately and then as a composite. Findings were similar and thus we report findings using the composite structure variable.

**Mediation Analyses**

We conducted secondary mediation analyses for those situations where the since last survey parenting variable had a significant effect on the current arousal variable (a path) and the current arousal variable had a significant effect on next timepoint SU or externalizing symptoms, in the same direction. This was the case for two situations- 1. Since last survey negative parenting to current negative arousal to next timepoint externalizing symptoms and 2. Since last survey low parental warmth to current negative arousal to next timepoint externalizing symptoms.

For these, to test mediation, following Baron & Kenny (1986), we conducted two sets of models. We first conducted two MLMs (one for negative parenting, one for warmth) to formally test the mediation a path (since last survey parenting to negative emotion) (see Table S1 for results of these models). We then conducted two MLMs (one for negative parenting, one for warmth) to formally test the b path (negative emotion to next timepoint externalizing symptoms, controlling for the effect of parenting) (see Table S2). In these models, the b paths were significant, supporting mediation. However, the c’ paths from parenting to externalizing symptoms in the presence of the mediator were still significant, suggesting that there was not “full” mediation.

We then calculated a Sobel test to test for mediation, using https://quantpsy.org/sobel/sobel.htm. The Sobel test for negative parenting was significant, supporting partial mediation, Sobel test statistic = 2.98, SE = .003, p = .003. For warmth, the Sobel test was a non-significant trend, Sobel test statistic = -1.90, SE = .001, p = .06. This suggests partial mediation from negative parenting to higher lagged externalizing symptoms through higher negative emotional arousal.

**Table S1**

*Parenting Associations with Adolescent Arousal (Mediation A Path)*

|  | Negative Emotion Arousal | | |
| --- | --- | --- | --- |
|  | b | *SE* | *p* |
| Negative Parenting Models |  |  |  |
| Intercept | 21.48 | 1.11 | <.001 |
| Weekend Status | -0.94 | 0.36 | .01 |
| Study Day | 0.03 | 0.02 | .20 |
| Neg Parenting (between-person) | 1.01 | 0.13 | <.001 |
| **Neg Parenting (within-person)** | **0.29** | **0.01** | **<.001** |
| Parental Warmth Models |  |  |  |
| Intercept | 21.15 | 1.46 | <.001 |
| Weekend Status | -0.79 | 0.37 | .03 |
| Study Day | 0.02 | 0.02 | .50 |
| Warmth (between-person) | -0.03 | 0.07 | .63 |
| **Warmth (within-person)** | **-0.06** | **0.01** | **<.001** |

*Note*. Neg = Negative. Bold indicates the mediation a path.

**Table S2**

*Negative Arousal Predicting Externalizing Symptoms, Controlling for Parenting (Mediation B Path)*

|  | Externalizing Symptoms | | |
| --- | --- | --- | --- |
|  | b | *SE* | *p* |
| Negative Parenting Model |  |  |  |
| Intercept | 10.08 | 0.54 | <.001 |
| Weekend Status | -0.004 | 0.20 | .98 |
| Study Day | -0.01 | 0.01 | .40 |
| Negative Parenting Recent (between-person) | 0.81 | 0.08 | <.001 |
| Negative Parenting Recent (within-person, concurrent) | 0.29 | 0.01 | <.001 |
| Negative Parenting Recent (within-person, lagged) | 0.03 | 0.01 | .001 |
| Negative Emotion Current (between-person) | 0.09 | 0.05 | .08 |
| Negative Emotion Current (within-person, concurrent) | 0.03 | 0.01 | <.001 |
| **Negative Emotion Current (within-person, lagged)** | **0.03** | **0.01** | **<.001** |
| Parental Warmth Model |  |  |  |
| Intercept | 9.93 | 0.87 | <.001 |
| Weekend Status | 0.14 | 0.04 | .26 |
| Study Day | -0.03 | 0.01 | .06 |
| Warmth Recent (between-person) | -0.04 | 0.04 | .33 |
| Warmth Recent (within-person, concurrent) | 0.01 | 0.01 | .26 |
| Warmth Recent (within-person, lagged) | -.02 | .01 | .01 |
| Negative Emotion Current (between-person) | 0.40 | 0.07 | <.001 |
| Negative Emotion Current (within-person, concurrent) | 0.07 | 0.01 | <.001 |
| **Negative Emotion Current (within-person, lagged)** | **0.02** | **0.01** | **.001** |

*Note.* Bold indicates the mediation b path.

**Supplement References**

Baron, R. M., & Kenny, D. A. (1986). The moderator–mediator variable distinction in social psychological research: Conceptual, strategic, and statistical considerations. *Journal of Personality and Social Psychology, 51*, 1173–1182. [https://doi.org/10.1037/0022-3514.51.6.1173](https://psycnet.apa.org/doi/10.1037/0022-3514.51.6.1173)

Tomko, R. L., Solhan, M. B., Carpenter, R. W., Brown, W. C., Jahng, S., Wood, P. K., & Trull, T. J. (2014). Measuring impulsivity in daily life: The momentary impulsivity scale. *Psychological Assessment*, *26*, 339-349.
